# Potential of Grasses in Phytolith Production in Soils Contaminated with Cadmium

**DOI:** 10.3390/plants9010109

**Published:** 2020-01-15

**Authors:** Múcio Mágno de Melo Farnezi, Enilson de Barros Silva, Lauana Lopes dos Santos, Alexandre Christofaro Silva, Paulo Henrique Grazziotti, Jeissica Taline Prochnow, Israel Marinho Pereira, Ivan da Costa Ilhéu Fontan

**Affiliations:** 1 Federal University of the Jequitinhonha and Mucuri Valley (UFVJM), Campus JK, Diamantina 39.100-000, Minas Gerais, Brazil; muciomagno@yahoo.com.br (M.M.d.M.F.); lauanasantos@ymail.com (L.L.d.S.); alexandre.christo@ufvjm.edu.br (A.C.S.); grazziot@yahoo.com.br (P.H.G.); jeissicataline@hotmail.com (J.T.P.); imarinhopereira@gmail.com (I.M.P.); 2 Federal Institute of Minas Gerais - Campus São João Evangelista, Av. Primeiro de Junho, 1043, Centro, São João Evangelista 39.705-000, Minas Gerais, Brazil; ivan.fontan@ifmg.edu.br

**Keywords:** heavy metal, *Urochloa decumbens*, *Urochloa brizantha*, *Megathyrsus maximus*, phytolith-occluded, Entisol, Oxisol

## Abstract

Cadmium (Cd) is a very toxic heavy metal occurring in places with anthropogenic activities, making it one of the most important environmental pollutants. Phytoremediation plants are used for recovery of metal-contaminated soils by their ability to absorb and tolerate high concentrations of heavy metals. This paper aims to evaluate the potential of grasses in phytolith production in soils contaminated with Cd. The experiments, separated by soil types (Typic Quartzipsamment, Xanthic Hapludox and Rhodic Hapludox), were conducted in a completely randomized design with a distribution of treatments in a 3 × 4 factorial scheme with three replications. The factors were three grasses (*Urochloa decumbens, Urochloa brizantha and* *Megathyrsus maximus*) and four concentrations of Cd applied in soils (0, 2, 4 and 12 mg kg^−1^). Grass growth decreased and increased Cd concentration in shoots of grasses with the increased Cd rates in soils. The toxic effect of Cd resulted in production and Cd occlusion in phytoliths produced in shoots of the grasses. Grasses showed potential for phytolith production, independent of soil type, providing phytoextraction of Cd in phytoliths. *Megathyrsus maximus* was the grass with the highest tolerance to Cd, evidenced by higher production and Cd capture in phytoliths for the evaluated soils. Phytolith production by grasses in Cd-contaminated soils is related to genetic and physiological differences of the evaluated grasses and Cd availability in soils.

## 1. Introduction

Cadmium (Cd) is an unessential element with elevated mobility and water solubility, being easily uptaken and accumulated in plants [1]. Moreover, Cd is a very toxic heavy metal with high environmental contamination capacity, especially where high anthropogenic activities occur [2]. Cd enters the environment mainly through industrial and mining activities, combined with improper application of chemical fertilizer and sewage sludge in soil [1,2].

Due to the toxicity potential and high persistence of Cd, Cd-polluted soils create an environmental problem that threatens animal, plant and human health. The toxicity and high persistence of Cd carry to soil contamination, causing an environmental problem that threatens plant, animal and human health [1,3]. Many studies have been conducted to decrease soil bioavailability of Cd in contaminated areas [4,5,6,7] to increase plant growth and yield for the purpose of safe food production. Cadmium is extremely toxic at concentrations between 5 and 10 mg kg^−1^ in dry mass for most plants [4,8] and can cause various biochemical, structural and physiological changes [1,2,4,9,10,11], as well as imbalance of plant nutritional status [8,12,13,14,15,16,17]. Plants develop various mechanisms of tolerance to metal accumulation in the shoots [4,14,18,19,20], being the occlusion and sequestration of Cd in phytolith produced in the shoots [1,10,14].

Phytoremediation technique has been used in order to restore heavy-metal contaminated soils [11]. This technique uses low-cost and ecofriendly technology, utilizing the potential of some plants to tolerate toxic soil elements [10]. The potential of a plant species in phytoremediation of metal-contaminated soils can be assessed in different ways; depending on the criteria, a plant may or may not be considered a phytoremediator of metal. Thus, indicator plants accumulate metals in their tissues and generally reflect levels of metals in soil [18]. Hyperaccumulator plants are those capable of extracting and accumulating the Cd concentration in tissue at values greater than 100 mg kg^−1^ dry mass [4,19]. Grasses have high potential for phytoremediation purposes due to desirable phytoextraction characteristics such as high-growth rate, biomass production, root growth and capacity to tolerate and accumulate toxic metal [15,19,21,22] with high phytolith production capacity [23,24,25,26].

Phytoliths are particles of amorphous silica with sizes ranging from 1 to 250 µm [27,28], formed by silicic acid-polymerization processes and absorbed from the soil solution by plant roots, which makes amorphous silica precipitates along with metals in cell walls, intercellular spaces or cell lumen [6,23,26,29]. The phytolith production can trap and neutralize harmful metal ions in some parts of plant tissues, increasing resistance against stresses caused by metals, especially for the Cyperaceae and Poaceae families [5,25,26,30]. Phytolith production reduces soil-soluble heavy metals with decreased risk of trophic-chain contamination due to their stability [1,2,14,17].

The production and heavy-metal occlusion in phytolith by plants is still not fully understood, particularly in Cd-contaminated soil. Phytolith production is influenced by phylogenetic and phenological characteristics, as well as soil and its elemental and mineralogical composition [5]. This paper aims to evaluate the potential of grasses in phytolith production in soils contaminated with Cd.

## 2. Results and Discussion

### 2.1. Cadmium Effects on Grass Biomass and Phytolith Production

**Figure 1:**
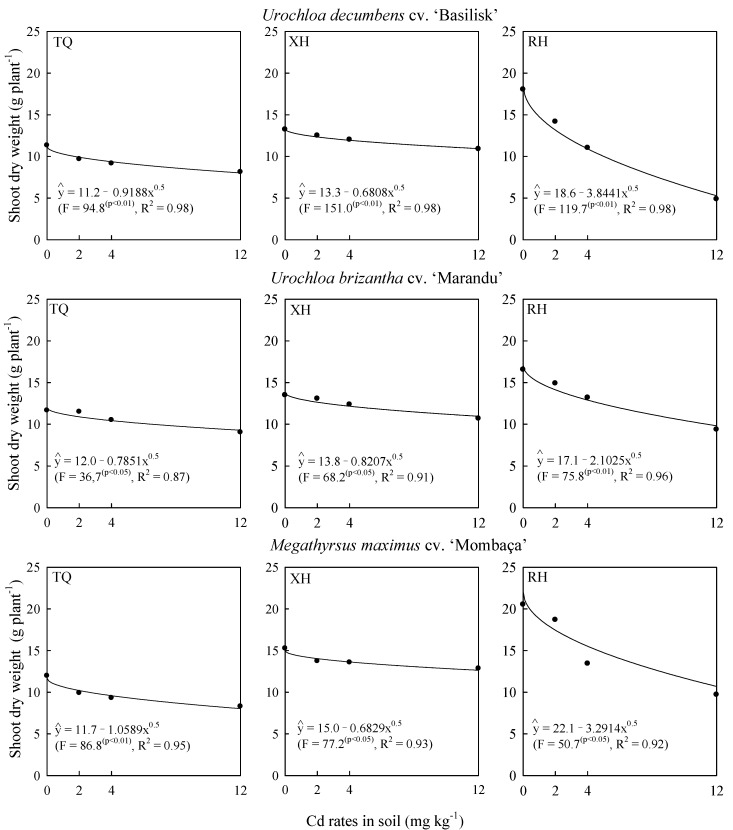
Shoot dry weight of grasses due to Cd rates in soil at 120 days after thinning in three soils. TQ: Typic Quartzipsamment. XH: Xantic Hapludox. RH: Rhodic Hapludox.

Shoot dry weight of grasses decreased with increasing Cd rates (*p* < 0.01) in all evaluated soils (Figure 1). The Cd supply even at low rates reduced the shoot dry weight of grasses in the evaluated soils (Figure 1), proving the effect of Cd phytotoxicity on plants [19]. Cadmium at low soil concentrations can interfere with photosynthesis and respiration processes, causing biochemical, morphological and physiological imbalances in plants [1,2,4,10,11]. In addition, Cd can cause alteration in nutrient concentration in plants due to interaction with cationic nutrients such as Ca, Mg, Cu, Fe, Mn and Zn [12,13,16,17], causing reduced biomass production. Based on linear regression coefficients (Figure 1), grasses tolerated more soil Cd when cultivated in Typic Quartzipsamment (TQ) and Xantic Hapludox (XH) soils than when cultivated in Rhodic Hapludox (RH). Higher Cd tolerance of grasses in sandy soils (TQ and RH) indicates that plant-growth capacity in soils with high Cd availability [4,5,6,7] relates to the ability of roots to exclude Cd from tissues, with the ability to chelate metal into a nontoxic compound or to inactivate in nonvital cell compartments [4].

**Figure 2:**
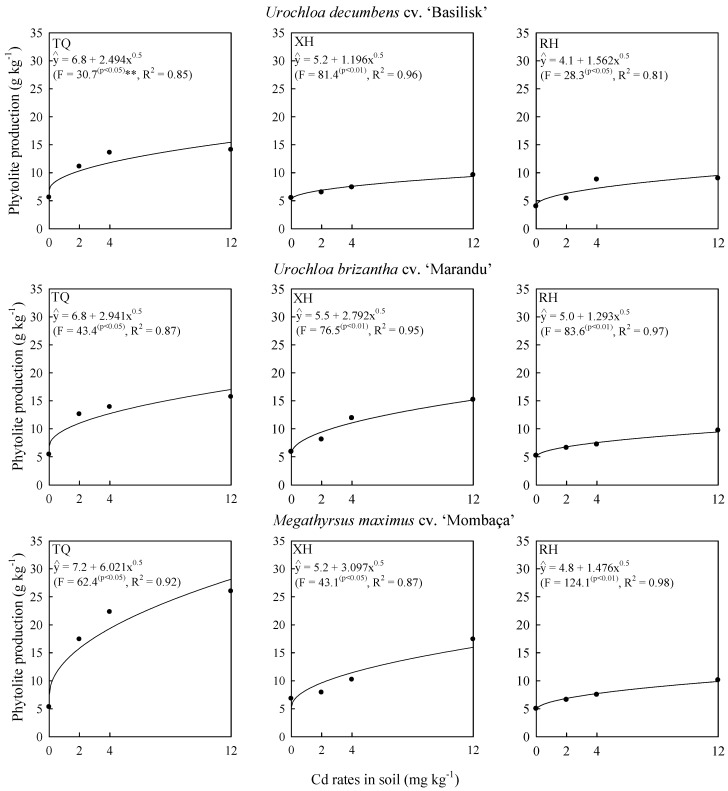
Phytolith production in shoots of grasses due to Cd rates in soil at 120 days after thinning in three soils. TQ: Typic Quartzipsamment. XH: Xantic Hapludox. RH: Rhodic Hapludox.

Grass tolerance may be related to the production of phytoliths, since, regardless of soil type and Cd rate applied, the three evaluated grasses presented potential for phytolith production (Figure 2). Several studies report that Poaceae species are large phytolith producers [3,15,23,24,26,27]. In addition, phytolith production in plant organs can be influenced by soil element availability [4,5,6,7]. The higher Cd concentration and availability in soil may reflect higher phytolith production, as observed in the present study, especially in grasses cultivated in TQ (Figure 2). Accordingly, sandy soils with low organic matter concentration and pH have higher Cd availability [4,7], and consequently provide higher phytolith production.

However, *Megathyrsus maximus* produced the largest amount of phytolith when cultivated in the three soils (Figure 2), indicating that plant phytolith production depends not only on soil, but also on genetic differences between plant species [5]. Phytolith concentration in plants varies widely, around 1 to 100 g kg^−1^ of their dry weight [30]. The phytolith concentration in shoots varied between 4.1 and 7.2 g kg^−1^ without application of Cd, and between 9.3 and 28.2 g kg^−1^ at the maximum Cd rate, and the highest values were found in *Megathyrsus maximus* cultivated in TQ (Figure 2). Gymnosperms generally accumulate less phytolith than angiosperms and other monocotyledons, and commonly accumulate fewer phytoliths than Poaceaes and Cyperaceaes, which are considered large producers, with about 150 g kg^−1^ of the dry weight of plants [24,25,26].

Sandy soils present characteristics such as low Fe and Mn concentration, lower cation-exchange capacity and clay concentration, associated with low pH values (<6.5) that tend to have higher Cd and Si availability, which may directly reflect the production and Cd capture by phytoliths [5]. Phytoliths are the main Si deposits in plant cells after uptake of dissolved Si(OH) or HSiO from soil solution [30]. Phytolith plants have diverse and multifunctional roles, especially in adverse soil conditions [17,30], such as soil contaminated with Cd.

### 2.2. Cadmium Concentration in Shoot and Phytolith

**Figure 3:**
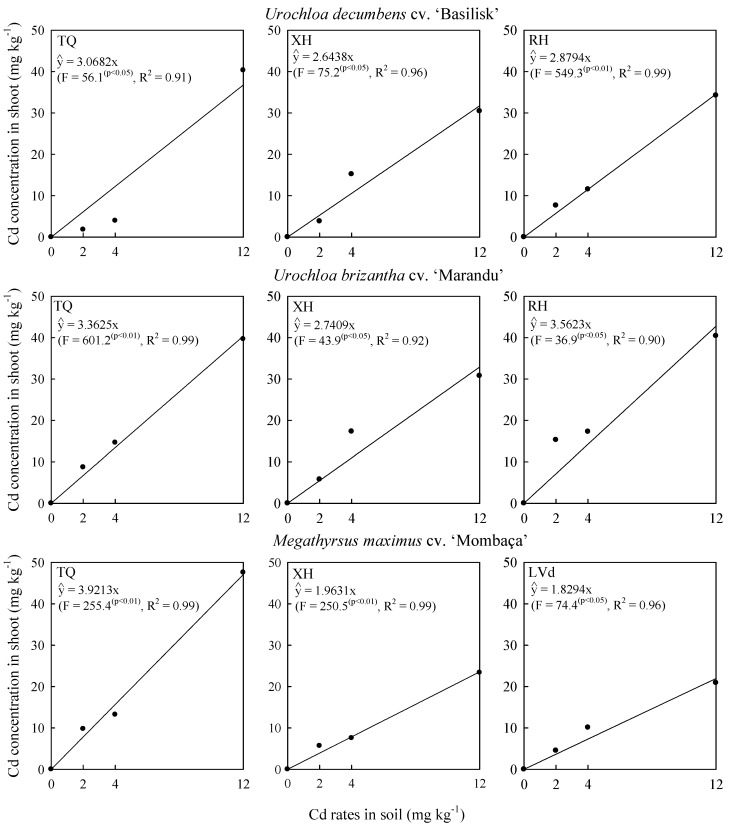
Cd concentration in shoots of grasses due to Cd rates in soil at 120 days after thinning in three soils. TQ: Typic Quartzipsamment. XH: Xantic Hapludox. RH: Rhodic Hapludox.

Cadmium concentrations in grass shoots increased linearly as Cd rates increased in the three soils (*p* < 0.01) (Figure 3), confirming the results found by [15,22]. Cd concentrations in shoots are higher in plants grown under higher Cd availability [4,7], where *Megathyrsus maximus* presented the highest Cd concentration in shoots when cultivated in TQ, and *Urochloa brizantha* when cultivated in Xantic Hapludox (XH) and RH (Figure 3).

Cadmium accumulation patterns in plants vary in tolerant, nontolerant and hyperaccumulating species [4,9]. Metal hyperaccumulating plants have high concentrations of Cd in the dry mass but produce little biomass, which results in low metal absorption per area [15,20].

**Figure 4:**
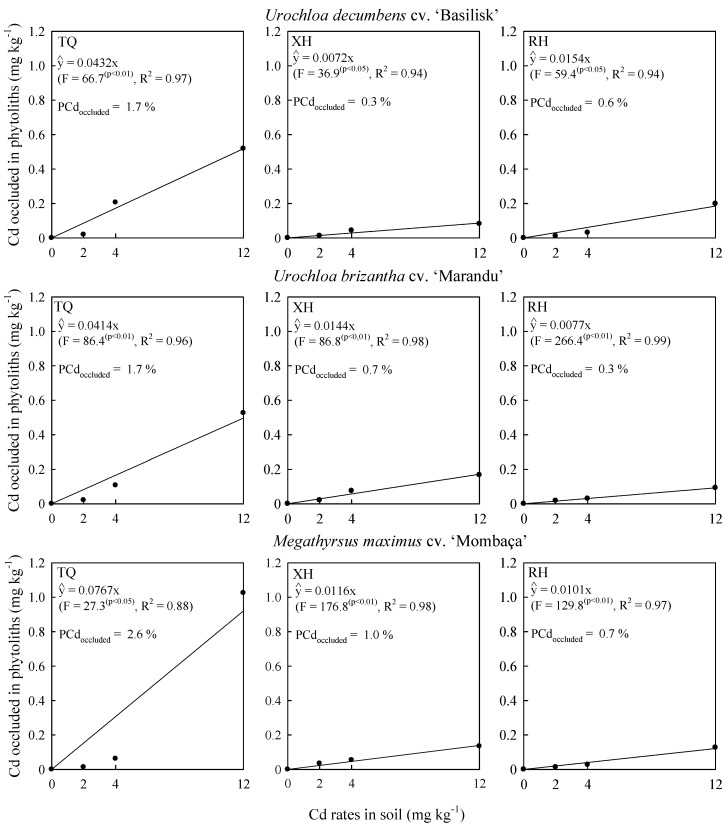
Cd occluded in phytoliths in shoots of grasses due to Cd rates in soil and the percentage of Cd occluded (PCd_occluded_) at 120 days after thinning in three soils. TQ: Typic Quartzipsamment. XH: Xantic Hapludox. RH: Rhodic Hapludox.

However, the evaluated grasses are not considered Cd-hyperaccumulating plants due to their low capacity to accumulate Cd (Figure 3) above 100 mg kg^−1^ Cd in dry mass [4,19] without presenting toxicity. The evaluated grasses can be classified as phytoextractors and/or bioindicators [18] due to their ability to extract Cd from the soil, not limiting the uptake and accumulation of Cd in shoots with increasing Cd rates (Figure 3). Thus, Cd is extracted from the soil by the grasses and, to a certain extent, stabilized in shoots, through its occlusion and/or sequestration during the phytolith production (Figure 4).

The different behaviors of species with respect to Cd concentrations in shoots (Figure 3) indicate that *Megathyrsus maximus* absorbs more Cd in sandy soil (TQ) while *Urochloa brizantha* absorbs more Cd in more clayey soils (XH and RH), which is reflected in the higher Cd concentration of shoots (Figure 3) and the reduction of biomass (Figure 1). The higher tolerance of *Megathyrsus maximus* cultivated in TQ and *Urochloa brizantha* in XH and RH confirm that the genetic and physiological differences [1,2,4,9,10,11] between the evaluated grasses, together with the physical, chemical and mineralogical soil attributes [4,5,6,7] may cause different tolerances to Cd.

Regardless of soil, phytolith produced by grasses was able to capture the metal Cd (Figure 2 and Figure 4). Cadmium concentrations in phytoliths showed the same tendency as Cd concentration in grass shoots (Figure 3), with linear increase due to increased Cd rates applied in soils (Figure 4). The evaluated grasses have a mechanism that allows the capture and accumulation of Cd (Figure 4). Cd capture and accumulation in phytoliths may be related to some defense mechanism of these grasses, which may help the species reduce Cd toxicity [14].

The grasses cultivated in the TQ reduced the shoot dry weight (Figure 1) and increased the phytolith production (Figure 2), the Cd concentration in shoots (Figure 3) and phytoliths (Figure 4), with increasing Cd rates applied to the soil. The observed trend reflects the Si effect on rice translocation and Cd toxicity; when synchronized Si and Cd accumulation occurs on the surface and within phytoliths, it reduces the potential risks of Cd contamination in rice [14].

The production and Cd occlusion in phytolith of grasses were higher because of the greater availability of Si (Table 1) and Cd (Figure 3) with higher percentage of Cd occlusion in phytoliths in shoots of grasses in TQ than in the XH and RH soils (Figure 4). Cd availability for plants is higher in acidic, sandy and low organic matter soils [4,7], where metal concentrations in the shoots and occlusion in phytoliths are directly related to metal concentrations and availability in soils [4,5,6,7]. In addition, there is a correlation in the distribution of Cd and Si, since Cd is usually deposited where Si is intensively deposited [14], and Si–Cd precipitation occurs [6,23,26]. Thus, the production of Cd-occluded phytoliths [14] may be one of the possible mechanisms for reducing Cd toxicity in the evaluated grasses.

The highest percentage of Cd concentration in phytoliths in the shoots presented by *Megathyrsus maximus* in the evaluated soils (Figure 4) indicates that this grass has a higher potential for Cd occlusion and consequently greater tolerance to Cd toxicity when compared to the other grasses, becoming a potential grass for phytoremediation of Cd. Moreover, this greater Cd occlusion in phytoliths (Figure 4) and possible tolerance may reflect the nutritional requirement of *Megathyrsus maximus*. Plants in better nutritional status are more tolerant to adverse growth factors [8,22], and *Megathyrsus maximus* has a higher production potential compared to *Urochloa* sp., being more responsive to soil fertilization [21]. However, production and elemental composition of phytoliths are influenced by element availability and absorption, climatic conditions (transpiration flow), plant species, silicon concentration and soil type, variety, location, disease resistance and fertilizer requirements [5,6,20,28,29].

## 3. Materials and Methods

### 3.1. Experimental Conditions

Three greenhouse experiments were performed in Diamantina, Brazil (18°15′ S, 43°36′ W, 1250 m a.s.l.). The experiments were conducted in a completely randomized design with three replications arranged in a 3 × 4 factorial scheme. The factors were three grass species (*Urochloa decumbens* (Stapf) R.D. Webster cv. Basilisk, *Urochloa brizantha* (Hochst. ex A. Rich.) R.D. Webster cv. Marandu and *Megathyrsus maximus* (Jacq.) B.K. Simon and S.W.L. Jacobs cv. Mombaça) and four Cd rates applied in soil (0, 2, 4 and 12 mg kg^−1^) conducted on three soil types. The Cd rates were based on the agricultural intervention values for soil established in São Paulo State, Brazil [31].

The soils were a Typic Quartzipsamment (TQ) (Arenosols in World Reference Base classification), a Xantic Hapludox (XH) and Rhodic Hapludox (RH) (Ferralsols in World Reference Base classification), classified according to Soil Taxonomy [32] and collected at 0.2 m depth. A subsample was collected, air dried and sieved at 2.0 mm for chemical and soil texture analysis [33] (Table 1). Total Cd concentration in soils was determined by U.S. Environmental Protection Agency (USEPA) 3052 method with microwave oven digestion [34] (Table 1). Cadmium analysis in soils was controlled with certified soil National Institute of Standards and Technology (NIST) Standard Reference Materials (SRM) 2709 San Joaquin soil and blank reagents. Total Si concentration was determined by X-ray fluorescence (Table 1).

Liming in soils was carried out with dolomitic limestone of 100% total neutralizing power to correct base saturation at 45%. Liming requirement (LR) was calculated by the formula LR (Mg ha^−1^) = (V_2_ − V_1_)CEC/100, where V_2_ is the recommended base saturation for grasses (45%) and V_1_ is the base saturation of soil analysis (Table 1). Lime reaction in soils occurred for 30 days with soil moisture at field capacity. Soil moisture was controlled by daily weighing throughout the experimental period.

Fertilization rates were 100 mg N (ammonium sulfate, urea), 150 mg K (potassium chloride), 50 mg S (ammonium sulfate), 1.0 mg B (boric acid), 1.5 mg Cu (copper dichloride), 5.0 mg Fe (ferrous chloride EDTA), 4.0 mg Mn (manganese dichloride) and 5.0 mg Zn (zinc chloride) per kg of soil. Phosphate fertilization was stipulated by the maximum phosphorus-adsorption capacity of each soil using the Langmuir second isotherm adsorption region [35]. Thus, P rate applied was 200 mg for TQ, 350 mg for XH and 450 mg for RH per kg of soil with source NaH_2_PO_4_. Fertilizer was mixed with the soil as a chemical reagent with an incubation period of 15 days. Cd rates were applied as pure reagent cadmium chloride after liming and fertilization with an incubation period of 15 days.

Grass sowing was performed in pots of polyvinyl chloride with an inside diameter of 0.18 m and a height of 0.20 m, with 3 kg of soil. Seedling thinning was performed at 14 days after grass emergence with evaluation of one plant per pot. Nitrogen fertilization in top-dressing for grasses was split into four applications of 50 mg kg^−1^ (urea) at 15-day intervals after thinning of grasses. Grasses were grown in a greenhouse under natural photoperiod, maximum/minimum air temperatures of 21–18 and 21–18 °C at night and day, respectively, and relative humidity between 55% and 78%.

### 3.2. Measurements

Grasses were harvested after 120 days of thinning seedlings. Shoot samples were packed in paper bags and oven-dried with forced-air circulation at 65 °C to a constant weight. Dry plant material was weighed on an analytical balance to measure the shoot dry weight. Materials were ground and subjected to nitroperchloric digestion (nitric acid (65% *v*/*v*) and perchloric acid (70% *v*/*v*), 2:1 ratio). Cd concentration was determined by graphite-furnace atomic absorption spectroscopy. Cadmium analysis in plant material was controlled with certified reference material NIST SRM 1573a tomato and blank reagents. Samples were reanalyzed if the determination of the NIST standard did not remain within 10% of certified values.

Phytolith concentration in grass shoots was determined according to the methodology described by [36]. The Cd occluded in phytoliths was extracted using the USEPA 3052 method with microwave digestion [34]. Cd concentration in phytoliths was determined by graphite-furnace atomic absorption spectroscopy.

### 3.3. Statistics and Calculations

The variables were subjected to joint analysis of variance, which consisted of the study of Cd rates and grasses within each soil type. Means for soil types and grasses were compared by the Tukey test (*p* < 0.05). Regression equations were adjusted for Cd rates for each variable evaluated.

The percentage of Cd occluded (PCd_occluded_) in phytoliths of grass shoots for maximum Cd rate (12 mg kg^−1^) applied to soils was calculated by dividing the Cd concentration in phytoliths by Cd concentration in grass shoots multiplied by 100. Cd concentration values in phytoliths and grass shoots were estimated by substituting the maximum Cd rate (12 mg kg^−1^) in the equations that correlate Cd rates with these variables.

## 4. Conclusions

Phytolith production by grasses in Cd-contaminated soils is related to genetic and physiological differences of evaluated grasses and Cd availability in soils. *Megathyrsus maximus* may be a future grass for the technique of phytostabilization and revegetation of Cd-contaminated soils. However, studies to elucidate Cd sequestration in grasses by phytoliths are needed.

## Figures and Tables

**Table 1:** Chemical attributes and soil texture before greenhouse experiments.

Attribute	Unit	Soil		
		TQ	XH	RH
pH ^(a)^_water_	-	5.1	5.4	5.5
P ^(b)^	mg kg^−1^	0.2	0.1	0.2
K ^(b)^	mmol_c_ kg^−1^	0.4	0.1	0.2
Ca ^(c)^	mmol_c_ kg^−1^	6.7	4.50	8.1
Mg ^(c)^	mmol_c_ kg^−1^	3.5	1.8	3.9
Al ^(c)^	mmol_c_ kg^−1^	7.8	4.2	1.6
CEC ^(d)^	mmol_c_ kg^−1^	40.6	71.4	49.2
Organic carbon	g kg^−1^	3.5	5.8	5.2
Cd ^(e)^	mg kg^−1^	0.0	0.0	0.0
P max ^(f)^	mg kg^−1^	100	200	250
Si ^(g)^	mg kg^−1^	558	330	119
Sand ^(h)^	g kg^−1^	830	580	310
Loam ^(h)^	g kg^−1^	110	70	180
Clay ^(h)^	g kg^−1^	60	350	510

^(a)^ Soil: water, 1:2.5. ^(b)^ Mehlich-1 extractor. ^(c)^ KCl 1 mol L^−1^ extractor. ^(d)^ Cation-exchange capacity. ^(e)^ USEPA 3052 method. ^(f)^ Maximum P adsorption capacity. ^(g)^ X-ray fluorescence (XRF). ^(h)^ Pipette method. TQ: Typic Quartzipsamment. XH: Xantic Hapludox. RH: Rhodic Hapludox.
